# Supplementary material for: Causal Relationship between Aging and Anorexia Nervosa: A White-Matter-Microstructure-Mediated Mendelian Randomization Analysis
Source: Biomedicines. 2024 Aug 16;12(8):1874. doi: 10.3390/biomedicines12081874 (PMC11351342; doi:10.3390/biomedicines12081874)
Supplement: Supplementary file 1 [file biomedicines-12-01874-s001.zip › Supplementary Figure/Figure S4/Singe SNP plot of MR analysis for TL to ubm-b-1517.pdf]

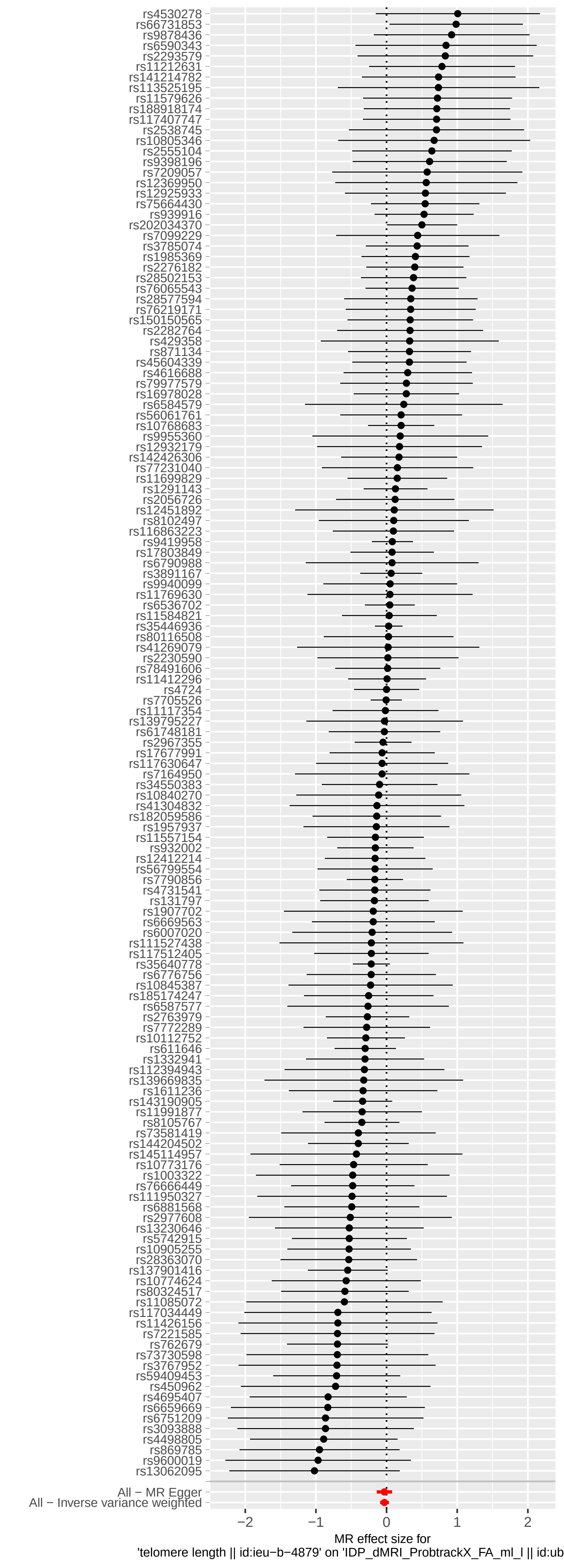
rs4530278
rs66731853
rs9878436
rs6590343
rs2293579
rs11212631
rs141214782
rs113525195
rs11579626
rs188918174
rs117407747
rs2538745
rs10805346
rs2555104
rs9398196
rs7209057
rs12369950
rs12925933
rs75664430
rs939916
rs202034370
rs7099229
rs3785074
rs1985369
rs2276182
rs28502153
rs76065543
rs28577594
rs76219171
rs150150565
rs2282764
rs429358
rs871134
rs45604339
rs4616688
rs79977579
rs16978028
rs6584579
rs56061761
rs10768683
rs9955360
rs12932179
rs142426306
rs77231040
rs11699829
rs1291143
rs2056726
rs12451892
rs8102497
rs116863223
rs9419958
rs17803849
rs6790988
rs3891167
rs9940099
rs11769630
rs6536702
rs11584821
rs35446936
rs80116508
rs41269079
rs2230590
rs78491606
rs11412296
rs4724
rs7705526
rs11117354
rs139795227
rs61748181
rs2967355
rs17677991
rs117630647
rs7164950
rs34550383
rs10840270
rs41304832
rs182059586
rs1957937
rs11557154
rs932002
rs12412214
rs56799554
rs7790856
rs4731541
rs131797
rs1907702
rs6669563
rs6007020
rs111527438
rs117512405
rs35640778
rs6776756
rs10845387
rs185174247
rs6587577
rs2763979
rs7772289
rs10112752
rs611646
rs1332941
rs112394943
rs139669835
rs1611236
rs143190905
rs11991877
rs8105767
rs73581419
rs144204502
rs145114957
rs10773176
rs1003322
rs76666449
rs111950327
rs6881568
rs2977608
rs13230646
rs5742915
rs10905255
rs28363070
rs137901416
rs10774624
rs80324517
rs11085072
rs117034449
rs11426156
rs7221585
rs762679
rs73730598
rs3767952
rs59409453
rs450962
rs4695407
rs6659669
rs6751209
rs3093888
rs4498805
rs869785
rs9600019
rs13062095
All − MR Egger
All − Inverse variance weighted
−2
−1
0
1
2
MR effect size for
'telomere length || id:ieu−b−4879' on 'IDP_dMRI_ProbtrackX_FA_ml_l || id:ub
